# Elements of Pathological Anatomy; Illustrated by Colored Engravings and 250 Woodcuts

**Published:** 1846-01

**Authors:** 


[TO BE CONTINUED.]

---

ART. V.—*Elements of Pathological Anatomy;* Illustrated by colored engravings and Two Hundred and Fifty Woodcuts. By SAMUEL D. GROSS, M.D., Professor of Surgery in the Medical Institute of Louisville; Late Professor of Pathological Anatomy in the Medical Department of the Cincinnati College; Surgeon to the Louisville Marine Hospital, etc., etc. *Second Edition, thoroughly Revised and greatly Enlarged.* Philadelphia: Ed. Barrington & Geo. D. Haswell. Louisville: James Maxwell, jr. 1845. Super-royal 8vo. pp. 822.

The words, "thoroughly revised, and greatly enlarged," however loosely applied in other instances, mean something on the present occasion. On comparing the former edition of this work with the present, it will be found not only that a large amount of matter, equal we are told to 300 pages, has been added, but that there is scarcely a page, or even a paragraph, which has escaped the "forked pursuit" of the author's pen, which has not been retouched, changed, condensed—in short, to revert again to the language of the title-page, *revised.* Indeed the labor expended on this revision, and in the collocation and arrangement of new facts, must have been little short of what would be required to write for the first time an entire volume. Evidently, though, it has been a labor of love, on the part of the author, rather than a task; and he was fortunate in securing publishers who seem to have entered into his own spirit. The mechanical execution of the work is deserving of the highest praise. The typography is beautiful. The illustrations, whether lithographs or xylographs, are, with very few exceptions, of the first order of merit, and many of them are really superb; the colored lithographs, especially, are superior to any we have ever seen from the press of this country. In short, as to all the externals, it is a volume of rare beauty and excellence, and one of which the publishers may well feel proud.

As in the first edition, Prof. Gross divides his treatise into—1. *General Pathological Anatomy*, wherein is discussed all that the lesions of the different organs and textures of the body have in common with each other; and 2. *Special Pathological Anatomy*, which presents us with the morbid anatomy of each particular organ and texture. The first part was noticed at some length in the second volume of this Journal (first series, 1840), and we should pass it over on the present occasion but for the fact that the author has added several new chapters which call for some notice, and that the doctrines which it contains not only lie at the very foundation of his system, but pervade every part and portion of it. In thus doing justice, however, to the author, we run the risk of doing injustice to the reader. We shall necessarily travel over ground familiar to the latter, point out to him that which was pointed out before, repeat to a greater or less extent that which has already been brought under his notice.

But we beg him to remember, that a really good story is never the worse for a second telling, nor a good book for a second reading—above all, that it is the repetition of error which becomes dangerous, not the repetition of truth.

As might be expected from the title, Prof. Gross deals very sparingly in speculation. He is disposed to stop where a good eye and a steady hand will no longer avail. He does not detain us by flickering and fanciful inquiries into the essence of disease. The solids and fluids of the body, he reminds us, are intimately blended and associated, have a mutual dependence, a reciprocal influence, and health seems to consist in a certain concert or harmony between the two. When this harmony is destroyed, when the fluid and solids are, as it were, arrayed against each other, when the latent sympathies which bind organ to organ, and texture to texture, are aroused and brought into play, we have *disease.* Disease, now, in the common acceptation, is either functional or organic. As used by most pathologists the word *organic* refers to *permanent* changes in the condition of an organ. But Prof. Gross means by it not only the permanent, but also every *temporary* alteration which the tissues or organs experience in a state of disease. As meaning the same thing, he employs occasionally the word structural. Used in this sense, it is exceedingly questionable if a functional disease exists, "a mere aberration of the physiological state of a part, without any change in its anatomical elements."

We come now to the two fundamental propositions of the author:

"Bearing in mind the above definition, it may be assumed, as a general proposition, liable to few exceptions, that all organic diseases, whatever be their seat or extent, are the result of inflammatory action, either of an acute or a chronic kind. To many, this proposition may be startling; nevertheless, if it be carefully examined, it will be found, I doubt not, to be grounded on fact. The truth of this remark will appear more evident as we proceed.

"The second proposition that may be stated is, that every inflammation, irritation, or morbid action, is originally of a local nature; that is to say, it always makes its impression in the first instance upon some particular part, texture, or organ. After this inflammation has continued for a longer or shorter period, it often happens that it extends to and implicates other structures. If, for instance, the mucous membrane of the stomach be fretted, the morbid action accruing from this cause will be confined at first to that lining; or, in more comprehensive terms, the disease will be strictly local in its character: by degrees, however, as the disorder progresses, the adjacent parts, such as the submucous cellular tissue, become affected; and, spreading still further, it next invades the muscular fibres of the organ, and, finally, the peritoneal covering. It is in this manner that most affections, which are originally local, extend their sphere of action so as to become general, whether they be considered simply in reference to one organ, to several, or to a great number of them."

Inflammation is greatly modified, as to intensity, progress, and mode of termination, by temperament, age, sex, habit, climate, season, the nature of the exciting cause, and the character of the tissue affected. The *period* of life most obnoxious to it is from the first to the tenth year. The skin, the mucous membranes, the lymphatic system, and the arachnoid membrane, are particularly liable to suffer in children; the lung and its coverings, the brain, liver, heart, the veins and arteries, in adults. The genital organs rarely suffer before puberty; but towards the decline of life, affections of the uterus, ovaries, breasts, and testicles, are frequent. Inflammation of the brain, spleen, heart, and arteries, is most common in *men;* that of the peritoneum, veins, and lymphatics in *women.* The *seasons* most favorable to it are winter and spring; it is more common in moist situations than dry, in cold and hot climates than in temperate. Inflammation varies in rapidity and intensity, and to express these differences our author retains the old terms *acute* and *chronic.* He objects to the word *sub-acute*, and employs instead the terms mild, slight, or moderate. Its progress, whether to reparation or the contrary, is much the most rapid in highly vascular and nervous organs. The affection may be *latent*, as in certain forms of pneumonia, arachnitis, and in typhoid fever. It may also be *intermittent*, as in some neuralgias, in chilblains, &c.

"The disease before us does not occur with equal frequency in all the organs and tissues of the body. There are some parts, in fact, in which it has been doubted, though, as I think, without any foundation in truth, whether this affection ever takes place. Such are the nails, the epidermis, and the hairs. These structures are supposed, by general anatomists, to be destitute of vessels, and, therefore, incapable of performing any vital action. This, however, is merely a conjecture; the fact remains to be proved, and, for my own part, I feel as certain that these textures are susceptible of inflammation, as that the liver is, the stomach, or any other organ.

"The cellular, mucous, serous, and dermoid textures are particularly prone to inflammation. In children, this is different. In them, the cutaneous and mucous textures are infinitely more liable to inflammation than the cellular tissue and serous membranes—the frequency of their attack being, as nearly as can be, in the order here stated. It is here that the disease can be studied with the greatest advantage, both as it respects its phenomena and modes of termination, inasmuch as it is usually well marked, intense in degree, and rapid in its progress. The synovial membranes, the fibrous envelopes, the bones, ligaments, and cartilages, with the muscles and their tendons, inflame with difficulty; but when the disease has once fastened itself upon them, they readily yield to its influence, the sufferings are often excessively severe, and the consequences very serious. The bloodvessels, nerves, and absorbents, are all more or less liable to phlegmasia. The conservative powers of these structures, especially of the former, is remarkable, and is strikingly evinced in cases of gangrene, where, as will be hereafter shown, they frequently retain their vitality amidst the half putrefied mass.

"Of the organs, some are more ready to take on inflammation than others. Those which are most frequently affected, at least in this country, are the lungs, spleen, liver, uterus, and brain. The heart, ovaries, thyroid body, pancreas, prostate gland, testicles, and kidneys, are comparatively rarely the seat of this disease.

"Respecting *specific* inflammation, some parts, again, are more prone to this disease than others. Thus, erysipelas commonly attacks the skin; anthrax, the subcutaneous cellular tissue; rheumatism, the fibrous envelopes of the extremities; tubercle, the lungs; scirrhus, the glandular organs. In the skin, numerous varieties of inflammation, both of an acute and chronic kind, are observed, that never occur in any other of the elementary textures and systems of the body. To this category specially belong the different species of eruptive and scaly diseases, which have their seat, for the most part, in the superficial portion of the dermis, the net-work of Malpighi, and the cuticle.

"Although we have employed here the term 'specific,' yet it must be confessed that it is one of very equivocal signification; and, in order to remove all ambiguity respecting it, it will be necessary to determine, if possible, the sense in which it is understood by professional men. Almost all surgical authors agree in stating that there are two descriptions of specific inflammation; one of which is produced, it is alleged, by a peculiar condition of the constitution, and the other by the action of a special virus. Under the influence of the first are developed what are denominated the heterologous formations, such as tubercle and scirrhus, together with gout, rheumatism, and erysipelas; under that of the latter a disease which manifests a particular train of phenomena, which exerts its effects only upon a particular set of structures, and which can only be excited by particular causes. To such a classification, surgically considered, there is no special objection; but, viewed philosophically, or in reference to the laws of healthy and abnormal action, there is no ground whatever for such a distinction. All morbid action, indeed, whether acute or chronic, is, properly speaking, specific, or, what is the same thing, exerts a particular influence in relation to the particular structure which it implicates, being accompanied by particular symptoms and particular products."—24, 25.

The author next proceeds to discuss the phenomena, seat, and especially that *questio vexata*—the nature of inflammation. Before entering upon these matters, however, we beg the reader's attention to the following passage:

"The *phenomena* usually enumerated as marking inflammation are redness, heat, pain, and swelling. These signs, however, are not constantly present, and, as might be supposed, they are liable to vary according to the nature of the exciting cause and the character of the part affected. But these are not the only circumstances which occur in inflammation: in every case there is a perversion of the vital actions, attended with an altered state of the nutritive and secretory functions. To affix to these several conditions their respective value, it will be necessary to allot to each of them a considerable share of attention. Most writers, until recently, attached too much importance to some of them, and too little to others; whilst they entirely overlooked the fact that they are always greatly modified by the nature of the tissue in which the malady, of which they are the indices, is located. If we regard the four phenomena, redness, heat, pain, and swelling, referred to above, as being essential to the process, it will be at once perceived that there can be few inflammations; and we shall therefore be obliged, in describing diseases, to invoke other names, such, as irritation and fever; a blind adherence to which has unfortunately tended too much to retard the progress of pathological science. Boerhaave enumerated one hundred and fifty varieties of fever: had he enumerated a thousand more he would have been much nearer to the truth, for he might then have specified nearly every form of inflammation, whether occurring in the external parts of the body, or in the interior organs. The word 'fever' is a conventional one, and is employed to designate, not the nature or seat of a disease, but simply the phenomena which it manifests. So also with the term irritation. Mr. Travers and others have written extensive treatises on this subject; but have they pointed out any thing concerning the essential character of this disease? have they told us anything of the peculiar condition of the nervous and vascular systems which accompanies it? So far as I am acquainted with their labors they have not: and yet men continue to talk about irritation, with its numerous varieties, as if they had the most perfect knowledge of its nature, seat, causes, and symptoms. A course such as this cannot but have a most dangerous tendency in practice; for what one physician describes as a fever, another will consider simply as an irritation, a third as an inflammation, and in this way no principles can ever be introduced as standards of treatment. The practice of medicine must continue to ebb and to flow with every tide of professional opinion.

"The time, however, cannot be far off, when the term fever must be entirely discarded from our books, and diseases named according to the tissues which they implicate. Then, and not till then, can it be expected that the laws of deranged action will be properly interpreted, or fully comprehended. All diseases, I feel confident, will ultimately be found to have a local origin and habitation; and if this should ever be proved to be true, the whole class of febrile maladies, with its hundred varieties and subdivisions, will cease to have a place in our medical treatise."—25, 26.

Without being as sanguine as the author, we still have a hope that his anticipations may be realized.

The *seat* of inflammation is in the capillary vessels. By this term we are to understand, not a set of vessels perfectly independent of the remainder of the vascular system, but "minute tubes which are every where interposed between the arteries and veins," communicating directly with both. Various opinions have been held concerning their structure. Many have supposed that they were membraneless; in other words, that the blood, after leaving the arteries, coursed hither and thither in the substance of the tissues, in channels formed out of the tissue itself. Bichat, and after him Beclard, taught that they were formed of a single coat continuous with the inner arterial and venous coat. Our author argues from analogy that the three tunics distinguished in the larger arteries and veins, are continued—excessively attenuated of course—into the capillary vessels. Recent investigations with the microscope have shown this to be the fact. Wedemayer states that they can readily be seen in vessels as large as the tenth of a line; Schwann discovered transverse fibres in the smallest capillaries of the mesentery of the frog; and Henle, Mueller, Baly, and others, have all seen the membranous walls, delicate but distinct, sometimes with oval bodies like epithelium-scales in their substance.

"Viewed in reference to their caliber, the capillaries are divisible into two classes. The one embraces those minute tubules which, though invisible to the naked eye, are found, when examined with the microscope, to be capable of carrying a continuous stream of blood, so as to give the part in which they are located a red appearance; the other includes those delicate vessels, the cavity of which is so small as to admit only a single globule at a time, and which it is extremely difficult to detect even with a good magnifier. By reflecting for a moment on the size of the red particles of the blood, estimated by most writers to be about the three thousandth part of an inch, the reader will be struck with the great tenuity of these vessels."—32.

The size of the capillaries is in general proportioned to that of the red globules of the blood. Their diameter appears to be uniform in the same organ, but varies in different organs from $\frac{1}{1000}$ to $\frac{1}{5000}$ of an inch, the mean being between $\frac{1}{3700}$ and $\frac{1}{1850}$ of an inch. The smallest, according to Weber, are found in the brain, where they are $\frac{1}{4700}$ of an inch. In the kidneys they are from $\frac{1}{2500}$ to $\frac{1}{1592}$; in the mucous membrane of the large intestine from $\frac{1}{2794}$ to $\frac{1}{1851}$; in the skin $\frac{1}{1150}$; in inflamed membrane from $\frac{1}{3840}$ to $\frac{1}{1923}$. In very young animals they are larger than in the adult, corresponding again to the red particles, which are larger in the former. They are smaller than any other tubes or ducts of the body. The biliary ducts, and the tubuli of the kidney, are several times larger than the capillary vessels. The primitive muscular and nervous fibres are a little smaller than the finest capillaries. These vessels are not equally numerous in all parts of the body.

"The parts which form the basis of the skeleton, together with the tendons, the cellular substance, the epidermis, nails, and hairs, have, comparatively, few of these vessels after the body has attained a certain degree of developement. In the early stages of life, however, most of these structures are highly vascular, and can be readily injected. The serous membranes appear to possess very few capillaries; in the healthy state, in fact, none can be discerned in them; yet, when inflamed, they are rendered highly vascular, and thousands of minute vessels, which before were invisible, are now perfectly distinct, giving the affected part a beautiful reddish aspect. The capillaries abound in the mucous membranes, the skin, the liver, spleen, lungs, and kidneys. They are also very numerous in the heart, the muscles of voluntary life, in the brain, and in the pia mater."—32.

The capillary vessels form a net-work in the meshes of which lies the proper substance of the organ or tissue. The meshes differ in size and shape in different parts. They are smallest in the lungs, and in the choroid membrane of the eye; they are likewise very close in the mucous membrane of the lungs, liver, and kidneys, and in the true skin. In the lung the interspaces are a little smaller than the capillaries; in the kidney, the mucous membranes, and the cutis vera, the diameter of the capillaries is to that of the interspaces as one to four, or one to three.

The capillaries, thus constituted and arranged, are important agents in nutrition, secretion, calorification, and absorption. Fluids pass into them and out from them, not through open extremities, which they have not; nor yet through *exhalants*, or visible openings in their parietes, of which they are equally destitute; but in consequence of the porosity which they possess in common with all animal membranes. They are pre-eminently endowed with nerves, which must, of necessity, be involved in any affections of the vessels. To show the joint agency of the nervous and vascular systems in the production of inflammation, the author refers to the experiments of Magendie, Brodie, and Philip. The former cut the fifth pair of nerves at the varolian bridge, in the rabbit, and saw it followed by inflammation of the surface of the eye. When the nerve was cut at the petrous portion of the temporal bone, the inflammation was still more violent, and resulted in total destruction of the eye. Division of the pneumo-gastric nerves was followed by engorgement of the lungs and the effusion of serosity into their substance, and by inflammation of the stomach. Brodie tied the brachial plexus of nerves, and the remote parts of the limb were attacked by inflammation, which resulted in gangrene.

In the author's view the first step in the process of inflammation is an altered sensibility of the part, produced by some hurtful agent. This local impression being reflected upon the cerebro-spinal axis, and through this upon the heart, the latter is incited to increased action, and more blood flows to the part concerned than it naturally receives, at the same time that the capillaries are dilated.

"The phenomena above alluded to, namely, the preternatural influx of blood, and the dilatation of the capillaries, can be easily detected by exciting irritation in the mensentery of a rabbit, the tale of a tadpole, the fin of a fish, or the web of a frog's foot, parts which are perfectly transparent, and therefore well calculated for the purpose. On viewing these structures with a microscope, in the sound state, numerous channels will be observed filled with blood, the red globules of which roll along in the most regular and beautiful order. If they be now irritated with spirits of wine, hot water, or diluted acid, the little rivulets just referred to, will be found to become dilated, from the manner in which the blood is crowded into them by the heart, which, in order to remove the local difficulty, is excited into sympathetic action. In a few minutes hundreds of vessels, which were previously invisible, will be seen shooting out in different directions, and connecting themselves with the sides of those that appeared in the first instance. These are not new channels, but old ones appertaining to the second class of capillaries, which are rendered evident by the intromission of red particles, which are either excluded in the healthy state, or pass along in so slow and gradual a manner as to elude the eye of the beholder. The little bodies which are thus introduced do not circulate, at first, with the same facility as in the other parts of the body; for, as the dilation of the little rivulets takes place by degrees, they have to force their way, and hence, after having advanced a short distance, they retreat slightly immediately after each pulsation of the heart, rebounding, as it were, upon each other. In this manner they travel on, surmounting every obstacle, until they finally reach the corresponding capillary veins, into which, as they are considerably more capacious, they rush, as into a vortex. * * * * *

"Such are the initial steps of inflammation. If the process be now checked by the removal of the exciting cause, the phenomena referred to gradually disappear, and the part recovers its natural tone and condition.

"If, on the other hand, the inflammation be allowed to proceed, another series of changes may be witnessed, surpassing, if possible, in point of interest, those just described.—The circulation now completely ceases; the blood assumes a dark modena color, and the coats of the vessels are rendered so soft as to be liable to give way on the slightest force. With these alterations the healthy functions of the part are suspended: it is red, hot, painful, and tumid; and its molecular intervals are filled with serosity, lymph, or pus. In this stage of the malady, the capillaries contain thick, viscid, partially clotted blood, which adheres with great tenacity to their inner surface, and opposes an effectual barrier to artificial injection, or to the removal of the fluid by pressure or ablution. * * Occasionally, again, the blood escapes from the vessels, and, forcing its way along the cellular tissue, forms new channels, through which it afterwards continues to circulate. This interesting phenomenon, which has been frequently noticed by Kaltenbrunner in the inflamed mesentery of the rabbit, is strictly analogous to what occurs in the organization of adventitious membranes, a subject to which the attention of the reader will be subsequently directed.

"Another striking phenomenon is the distended condition of the larger vessels leading to the inflamed part. When the disease is at its height, the congestion often extends to a considerable distance; the blood is unnaturally dark, thick, and viscid, and artificial injection is difficult, sometimes impracticable."—34, 35, 36.

This description of the changes in an inflamed part, though brief, is in the main correct. It is right, however, to observe, that the stagnation of blood is partial, occurring here and there in points only of the affected part, as if the *onus* of the inflammatory action fell more particularly on these points. It is not universal unless gangrene is imminent. It must be remembered, also, that this description answers to traumatic inflammation.

Let us go back now (for, to suit our own convenience, we have slightly changed the author's arrangement) to the phenomena usually enumerated as marking the occurrence of the changes just described. And first of the discoloration, which varies from the slightest rose to the deepest purple.

"There are are some tissues which naturally contain little blood, or which convey only serosity, and these, of course, are never much discolored when affected with disease. The tendons, ligaments, and cartilages are seldom reddened, no matter what may be the intensity of the inflammation. In the fibrous membranes, such as the pericardium, the dura mater, and sclerotic coat of the eye, the discoloration is usually of a lilac or purple hue, with a shade of blue. In the mucous lining of the alimentary tube, the redness, in the early stage of the disease, is bright and florid, like that of arterial blood; but, as it progresses, it often assumes a dark violet, or black appearance, especially when it is about to pass into gangrene. A striking exemplification of the truth of this remark is afforded by the mucous membrane of the fauces in the malignant form of scarlatina. In the beginning of this disease, the tonsils and adjacent parts are of a bright red, which is often, in the course of a few hours, converted into a deep purple. In the skin, the redness is sometimes of a scarlet color;at other times, it has a yellow tinge, with various shades of mahogany. The yellow color is most commonly witnessed along with derangement of the liver; hence the frequency of its occurrence in the latter stages of erysipelas and anthrax. In inflammation of the pleura and peritonæum, the redness is, at first, of a lilac hue; afterwards of a scarlet, brownish, or violet. In the arachnoid there is rarely, if ever, any perceptible discoloration.

"Inflammation of the spleen and liver is attended with a purple hue: when the brain is affected, the color is generally rosaceous, cineritious, or like the lees of wine. The salivary glands are usually of a pink complexion; the kidneys of a deep violet; the testicles and ovaries of a reddish yellow. In the lungs the color varies from the slightest rose to the deepest purple."—26.

The discoloration assumes various forms, as the arborescent, the capilliform, the punctuated, and the blotch-like. Sometimes, in the inner coat of the arteries for example, it is uniform, as if it were dyed into the part. In all cases it is owing to the unnatural influx of red globules, and is of a brighter or darker hue, according as the globular movement is quick or slow.

Hunter's experiments led him to believe there was no preternatural development of *heat* in inflamed structures. Subsequent investigations have shown that the reverse sometimes obtains. According to experiments made by the author, the average temperature of the blood in health is 96° (usually stated at 98°) whilst in disease it sometimes falls as low as 92°, or rises as high as 104°. The heat of the body is certainly subject to modifications. Home found the oviduct of a frog ready to spawn two degrees hotter than the heart; and, according to Dunglison, the uterus during labor may have a temperature of 104°. The doctrine of the day is, that animal heat is generated by the union of oxygen with carbon and hydrogen in respiration and the organic processes. Nevertheless, as our author remarks, it must be influenced by the nervous system, and hence any alteration in the innervation of a part would be attended by a change of heat, either *plus* or *minus*. In erysipelas, furuncle, and anthrax, the thermometer has been seen as high as 107°. In an inflamed scrofulous tumor Becquerel and Breschet found the temperature raised $5\frac{1}{4}$° above that of the body. In the ligaments, bones, cartilages, fibrous membranes, and tendons, the extrication of heat is slight.

*Pain*, one of the most important symptoms, varies, like redness, according to the nature of the part affected. It is most keenly felt in the parts most highly endowed with nerves and vessels. Bones, ligaments, tendons, cartilages, and fibrous membranes, do not evince much sensibility in the healthy state, but are exquisitely sensitive when inflamed. In the different viscera, inflammation of the coverings is far more painful than that of the proper structure. In inflammation of mucous membranes pain is sometimes excessive, sometimes absent; the ulcers in the ileum and colon during phthisis, afford an instance of the latter. Pain is much greater at the mucous outlets, where these membranes join the skin, and is owing to such parts being supplied with nerves from the cerebro-spinal instead of the ganglionic system. Pain varies in character: it is persistent, intermittent, or periodical; is sharp and lancinating in the pleura, acute and pulsatile in the cellular tissue, obtuse and gravative in the glandular organs, prurient and smarting in the skin, dull and gnawing in the bones, throbbing when pus is about to be formed.

*Swelling*, caused by engorgement of the vessels and the effusion of new matter—serum, lymph, pus, or blood—varies according to the vascularity and laxity of the part. It is well marked in the cellular substance, but is quite absent in the skin, ligaments, tendons, muscles, serous and fibrous textures, cartilages, bones, and in many of the viscera. In the mucous membranes, it occurs at the vulva, mouth of the larynx, and in the ocular conjunctiva, though here it is really in the submucous cellular tissue.

"From the hasty survey which has been taken of these symptoms, we are authorized to conclude that they are by no means entitled to the stress which has been generally placed upon them by writers. In many instances there is an entire absence of at least one or two of them, and yet the part is absolutely in a state of high inflammation. How often does it happen, that enteritis is lighted up, and goes on to destructive disorganization, without even the slightest indication of its presence! In arachnitis, the only symptom, frequently, is severe cephalalgia, with delirium and partial paralysis. The patient dies, and, on examination, the membrane is found to retain its natural thinness, and to be as free from injection as in the sound state. In such a case, should there be but little effusion of serum and fibrin, a superficial observer might conclude that there never had been any inflammation, or that what he saw was the result solely of irritation. The injurious tendency which such a mode of procedure would exert on the practice of medicine is too obvious to require any comment in this place. In reasoning on this subject, the physician should constantly bear in mind the important fact, that the symptoms which have been enumerated above, although they are frequently all present, are not necessarily so, and that the absence of some of them is not a sufficient proof that there does not exist inflammation. By such course alone can he expect to escape error."—29, 30.

But there are other symptoms still, insisted on by the author as attending all inflammations. "Perversion of the vital function" is rarely absent, and is generally greatest when inflammatory action is most severe, from which period it gradually subsides. Sometimes it is the only symptom present. Gastritis, for example, will be shown by nothing but frequent irritability; duodenitis, by arrest of chylification; hepatitis, by scanty and vitiated bile; nephritis, by the absence of urinary secretion. The function of absorption is also affected: strychnine in solution has been applied with impunity to an inflamed serous membrane, and prussic acid to the conjunctiva, the schneiderian membrane, and the mucous lining of the vagina. In other cases the alteration is not so extreme. Morphia applied to the skin inflamed by a blister tranquillizes the system; diluent drinks, such as gum water, moderately given, are absorbed by the inflamed stomach. Nutrition is likewise perverted: an inflamed organ becomes atrophied, the particles which nourish it being withheld; or the molecular arrangement is changed, there is an alteration of color and consistence—in other words, there is softening.

Leaving the subject of inflammation for a time, it is necessary to inquire into the causes capable of producing congestion and discoloration of the tissues "immediately prior to, during, or subsequently to, the extinction of life." The causes acting before death may be referred to mechanical obstructions to the free return of blood, and to the effects of stimulating agents taken as food, drink, medicine, or poison. The latter will be considered in another part of the work. As to the former, the obstruction may arise from a tumor, some morbid deposit, or the obliteration of a vessel; or, what is still more common, from organic lesion of the cardiac, the pulmonary, or the aortic valves, the ariculo-ventricular apertures, or the mouth of the pulmonary artery and aorta. All these are unquestionable causes of congestion, but, the congestion being permanent, it is by no means so clear, in the opinion of the author, that it does not induce actual inflammation.

"However this may be, it seems to me that pure, uncomplicated congestion, in whatever parts of the body occurring, must uniformly depend upon one or other of the following circumstances: 1. Obstruction of the heart and great vessels, by the formation of fibrinous concretions during the last struggles of life. 2. Partial paralysis of the heart, disqualifying it, to a greater or less extent, for carrying on the circulation. 3. Asphyxia, whether induced by actual strangulation, the inhalation of deleterious gases, or difficult dissolution in ordinary sickness."—37.

The congestion arising from the first two classes is generally partial, and limited to the more dependent portions of the body. That arising from asphyxia pervades the entire body, and is very conspicuous in the skin, lungs, heart, and great vessels, and where the skin and mucous membranes unite. In both cases the discoloration is of a dull bluish tint, and is more uniform than that of inflammation.

After death, discoloration may be produced by gravitation of the blood, and by its transudation, or that of some of its elements, through the walls of the vessels. The discoloration arising from the former cause (the *hypostatic hyperæmia* of the French school) is usually situated on the posterior parts of the body, but may be produced in any situation by placing the cadaver in a particular position. It is most conspicuous in organs where capillary vessels abound; and, as the subject usually lies, is observed in the posterior part of the lungs, liver, and kidneys, in the lobes of the cerebellum and those of the cerebrum, in the occipital portion of the cerebral coverings, and their continuation in the spinal canal, in the most dependent part of the stomach, and the folds of intestine which are most dependent, and in the skin on the back part of the neck, trunk, and extremities. The hue varies as in inflammation from slight rose to deep red, but is generally fainter and more uniform, and may be removed by pressure or ablution. It depends on dark-colored blood, generally fluid, sometimes partly coagulated, contained in the smaller veins, and these can generally be traced to larger vessels filled in the same way.

Discoloration from transudation is generally connected with the putrefactive process, and hence, unless in warm wet weather, is rarely observed until twenty-four hours have elapsed. The fluid (which is the serum and the coloring matter of the red globules rather than the blood itself) after percolating the vessels, is diffused among the surrounding tissues, rendering them unnaturally red and moist; or forms red bands, patches, streaks, points, or aborescent lines; or it may collect in the different serous cavities, as in the cerebral and spinal arachnoid, the peritonæum, or the pleura. The redness is generally of the scarlet hue; in the lining membrane of the heart, arteries, and veins, it is a mere scarlet stain, frequently uniform, sometimes figurate, and readily distinguishable from inflammation by the absence of other alterations. The arborescent redness is frequently observed in the stomach and bowels, where it corresponds to the large veins; the same appearance may be observed in the skin of the posterior parts of the body. This scarlet hue is at length lost in the muddy, brownish, and greenish appearances of advanced putrefaction—a change which takes place soonest in warm weather and in plethoric subjects. Another source of cadaveric coloration is exposure to atmospheric air. Warm weather and the direct solar rays are most favorable to its production; it is observed in the spleen, liver, kidneys, and heart, the inner arterial and venous coat, and the alimentary mucous membrane. The redness is of the bright arterial hue, and is diffused over a large surface. In the stomach and bowels it sometimes assumes the appearance of small florid specks. It is attributed to absorption of atmospheric oxygen, and, by immersion in a gentle stream of water or in dilute vinegar, is changed to a dark purple.

The physician should be able to distinguish the congestion and discoloration produced by these causes from those of inflammatory origin, but it is not always easy.

"Should there be merely some degree of redness, with ramiform injection, traceable to some large venous trunk, unaccompanied by effusion, softening, opacity, induration, thickening, or ulceration, the presumption is strong that these appearances are the result solely of congestion, produced by some one or more of the causes previously pointed out. If, on the other hand, the discoloration and vascularity are associated with some, or all, of the anatomical characters here indicated, it must be concluded that they are dependent upon inflammatory irritation, since they afford the best possible evidence of the existence of that lesion."—39.

After a glance at some of the hypotheses that have been put forth concerning the nature of inflammation, Dr. Gross proceeds to treat, in distinct chapters, of the different terminations of the process. In reference to *acute* inflammation these are, effusions of serum, lymph, pus, and blood, together with softening and gangrene. Except the last, these are in fact *conditions* of inflammation, not terminations, and indicate so many degrees of the process, constituting a sort of "phlegmasial scale." The chief conditions or terminations of *chronic* inflammation are, ulceration, granulation, cicatrization, and induration. Hemorrhage, softening, and gangrene, may likewise attend the chronic variety, but the occurrence is rare.

Chapter II—Is devoted to the *effusion of serum.* This is one of the most constant attendants of inflammation, whether simple or specific, acute or chronic, mild or intense. It is most frequently seen in the cellular tissue, in organs where that tissue is abundant, and in the serous cavities. The denser structures, as tendons, ligaments, aponeuroses, cartilages, and bones, the liver, kidney, womb, and prostate gland, afford very little. The brain, spinal chord, nerves and vessels, furnish it very sparingly. The mucous membranes in certain diseases pour out a large quantity; in the skin, it is observed in the vesications preceding gangrene, and in those consequent on certain irritants. It is likewise contained in hydatids and serous cysts.

The effusion is essentially the same in whatever organ or texture it occurs, and both in physical properties and chemical composition is analogous to the serum of the blood. Like it, it is sometimes as colorless as the purest rock-water; then it has various shades of yellow and red, from admixture with the coloring matter of the red globules and that of the bile. Occasionally it has a greenish tint, and that poured out by the peritonæum, in cases of strangulated intestine, is of the color of coffee-grounds. Like the serum of the blood, it consists of a large quantity of water (800 or 900 parts in 1,000) a variable quantity of albumen, some muco-extractive matter, muriate and sulphate of soda, phosphate of lime, iron, and magnesia. The quantity of albumen varies from eighty parts in a thousand to a mere trace. In the fluid of hydrocephalus and hydrorachitis, the amount is scarcely appreciable. In some instances it has been found to contain cholesterine and uric acid.

The specific gravity of the fluid is generally less than that of the serum of the blood. The quantity varies from a few ounces to many gallons. Sometimes it is poured out with great rapidity, as after the stings of certain insects, and the bites of venomous reptiles; and in certain cases of pleuritis.

Our author teaches not only that an effusion of serum is an accompaniment of all inflammations, but that it never takes place without inflammation. The serous effusions attending obliteration of the veins are generally adduced as examples arising from a purely mechanical cause. For example, if one of the femoral veins be obliterated, or compressed by a tumor, there is an œdematous condition of the corresponding extremity. If the obstruction exists in the ascending hollow vein, it will be followed by effusion in both extremities; if in the descending, by œdema of the upper half of the body. If the portal vein be obstructed or obliterated, the collection takes place in the peritonæum; if the obstacle exist in the heart itself, there is a general tendency to dropsical effusion in the whole body. Now copious accumulations of fluid are observed in many of the cavities where the local and general symptoms during life attest the presence of the inflammatory process, but where, after death, no increased vascularity, no patches or bands of lymph, nothing in fact but a little opacity, can be found. These instances are not unfrequent; and therefore the non-existence of inflammation must not be predicated of the absence of these symptoms.

"Such, then, being the difficulty of recognizing the presence of inflammation, where every symptom during life gives indubitable evidence of its existence, can it be wondered at that, in the instances above referred to, pathologists should still consider the effusion of serum as the result merely of mechanical obstruction? The question may now be asked, can such an obstruction exist, to any considerable extent, without producing a state of parts analagous to, if not really indentical with, inflammation? I would answer, no. Let it be supposed that the obstacle exists in the ascending hollow vein. This vessel is destined to return the blood from the inferior extremities, the pelvis and abdomen, to the right side of the heart. But, failing in the accomplishment of this object, from the difficulty adverted to, the blood is interrupted in its passage upwards, and congestion of all the vessels, both large and small, is the result. This congestion is not transient, but permanent; and it is scarcely reasonable to presume, judging from our knowledge of the circulation, that this state could exist long without producing an altered condition of the sensibility of the parts affected, attended with more or less redness, and effusion of serosity. The peritonæum and cellular tissue of the limbs are the structures which bear the onus of the obstruction, and these, it is well known, are parts which are most liberally supplied with serous capillaries. But, it may be said that the effusion may result from perverted action, from irritation, or disturbed function: all this may be true, and yet not in the least invalidate our position. Every body knows that in inflammation, there is perverted action, or deranged function, with irritation, or altered sensibility. These terms, therefore, if they mean any thing at all, only denote certain conditions—not the cause of these conditions; as redness, heat, pain, and turgescence are not inflammation, but only so many symptoms of it."—44.

A scirrhous tumor of the liver, situated so as to fret the serous covering, is attended by ascites, even though the portal circulation is free; so hydrothorax is induced by tubercles of the lungs, hydrocele follows upon carcinoma of the testicle, hydrocephalus succeeds heterologous growths of the brain. In these and similar cases the morbid deposit, in the opinion of our author, acts like a foreign substance, causing inflammation in the serous lining which results in an effusion of serum. C.

